# The Localisation of Higher Psychical Function

**Published:** 1906-02-24

**Authors:** 


### THE LOCALISATION OF HIGHER PSYCHICAL FUNCTION.

Drs. Charles Mills and T. H. Weisenburg,[1] in a paper dealing with this subject, and illustrated by a case recently under their observation, state that the conception of cerebral localisation of function dates from the beginning of the nineteenth century. Gall, the author of it, expressed his opinion not only that different portions of the brain were differently occupied, but that conclusions could be drawn as to the relative activity of the various parts in various individuals from a study of the external configuration of the skull. It is pointed out that although at a later stage Gall lost himself in the mazes of phrenology, the world is distinctly indebted to him for an idea which has since borne fruit of the greatest value. Three-quarters of a century had elapsed before the next advance of any moment in the direction of cerebral localisation. This occurred in 1870 when Broca identified the seat of the speech centre in the convolution which bears his name. In the succeeding decade the work of Hitzig and Fritsch, Ferrier and Charcot, based upon suggestions of Hughlings Jackson, definitely established the existence of motor areas upon the cortex. Lastly, in 1893, Flechsig, by means of embryological researches, demonstrated and localised certain association-centres for higher psychical functions.

The authors maintain that the highest mental faculties and functions in man have their material representation in the prefrontal lobes, and especially in the prefrontal lobe of the left side. They insist upon the necessity of distinction between what they term "fundamental psychical functions," such as attention, will, comparison, reasoning, and imagination, on the one hand, and emotions such as pride, vanity, and the rest on the other. They affirm that the former are definitely represented in special areas, while the latter find their representation in several parts of the brain at once.

In support of this thesis it is stated the examination of the brains of certain intellectually notable men has revealed a special development of the prefrontal lobes, while on the other hand the brains of criminals, paranoiacs, and low types generally, such as negroes, indicate a relatively deficient development of the area in question. The authors have found that in spite of the massive literature which has of late years grown up round the question of cerebral localisation, the record of associated psychical conditions is singularly meagre. Phelps, however, has collected no less than 800 instances of intracranial trauma. In three hundred of these cases examination of the brain was made either by operation or at autopsy, and the general conclusion of the study was to the effect that where mental symptoms had been observed, left frontal lesions were the rule, while lesions situated in other quarters of the brain were not associated with mental disturbances.

The authors' case in point was that of a doctor aged seventy-one, who, some eight months before his death, was observed to become altered in his manner. He lost his grip of work, and his memory for recent events showed signal evidence of impairment. Thus he would assert the existence of, and make money charges on account of, patients who had been long dead, and would go from place to place searching for them. He showed signs of mental confusion in the narration of events, and his disposition became altered to one of exaggerated mildness. Some five months before his death his power of writing began to fail, his letters being badly formed, and his use of words often confused. Autopsy showed that his left prefrontal lobe was the seat of a tumour which was accurately limited by this region, that is to say, the region in front of the speech centre.

In the discussion following the reading of the paper, Dr. House confirmed with reservations the thesis of the authors as to the prefrontal localisation of higher psychical function. He stated that in autopsies upon those who had died of general paralysis of the insane, it was common to find that when mental symptoms had preponderated during life, the evidences of cortical lesion were most marked in the prefrontal regions. He mentioned some cases from his own experience. The first was that of a man who had suffered a bullet wound of the frontal lobes. In this case there was no evidence of mental disturbance. In the second case a cyst of the right prefrontal lobe had been associated during life with weakness of the left side of the body accompanied by slow and confused cerebration. The balance of opinion was favourable to the contention advanced by the authors, although certain cases, such as the first one quoted by Dr. House, appear to preclude dogmatic conclusions.

[1] Jour. of Amer. Med. Assoc., Feb. 3, 1906.

